# Non-political anger shifts political preferences towards stronger leaders

**DOI:** 10.1038/s41598-022-15765-8

**Published:** 2022-07-11

**Authors:** Klaudia B. Ambroziak, Lou Safra, Manos Tsakiris

**Affiliations:** 1 The Warburg Institute, School of Advanced Study, University of London, London, UK; 2 Sciences Po, CEVIPOF, CNRS, Paris, France; 3 Department of Psychology, Royal Holloway University of London, London, UK; 4 Centre for the Politics of Feelings, School of Advanced Study, University of London, London, UK

**Keywords:** Psychology, Human behaviour

## Abstract

Past research has shown that anger is associated with support for confrontational and punitive responses during crises, and notably with the endorsement of authoritarian ideologies. One important question is whether it is anger generated specifically in a political context that explains the association between anger and specific political preferences or whether any feeling of anger would be associated with changes in political attitudes. Here, we tested the effect of non-politically motivated incidental anger on the preference for strong leaders. In line with past research, we predicted that anger would increase preferences for strong leaders. Across two experiments, we exposed participants to an anger induction task. Before and after this experimental manipulation, we measured participants’ political leader preferences by asking them to choose between the faces of two leaders they would vote for in a hypothetical election. The level of self-reported anger predicted the probability of choosing more dominant-looking and less trustworthy-looking leaders after the induction, suggesting that even non-political incidental anger increases preferences for strong leaders.

## Introduction

If we asked you to recall the last time you were angry, chances are that you would not have to look far into the past. In a classic study investigating anger^1^, it was found that most people reported being mildly to moderately angry ‘anywhere from several times a day to several times a week’. Besides being a very common emotion in everyday life, anger is not an epiphenomenon in politics and anger that is generated in a political context seems to have increased in recent years. While in 2012 less than half of American voters said they were angry at the other party’s presidential candidate, in 2016 this sentiment was shared by 72 percent of Democrats and 66 percent of Republican voters^2^. Similar trends are observed over a longer period of time (2008–2016) as documented in an analysis from the American National Election Studies and the Wesleyan Media Project that supports the observation that the US public is increasingly angry and that politicians often seek to elicit anger^3^.

In particular, anger has been shown to be a typical reaction to political crises^4–7^, and angry reactions to crises have important political consequences. Compared to other negatively valenced emotions such as fear and anxiety, during crises, anger is specifically associated with support for confrontational, aggressive responses e.g., support for expanding a war^7–11^. For instance, Lerner and colleagues (2003) showed that anger leads to lower estimates of risk of a terrorist attack as compared to fear and anxiety but at the same time increases the support for punitive actions in government policy, such as deporting foreigners. Recently, it was shown that people who were angry about the terrorist attack at Charlie Hebdo were more likely to support an authoritarian party (e.g. the French Front National) than people for whom the primary response to the attack was fear^7^. Similarly, Laustsen and Petersen in a study conducted in a real-world conflict setting^12^, during the Russian invasion of Crimea, found increased preference for dominant leaders only in participants for whom the primary response to conflict was anger, not fear, suggesting that the emotional response to the external stressor affects people's political judgements and preferences.

Even though anger is recognized as having a large impact on political attitudes, it remains unclear whether anger should be generated specifically in a political context to affect political attitudes or if non-politically specific anger would also influence political behaviour. Indeed, the literature in experimental psychology reveals that incidental anger affects people’s judgements, suggesting that even non-politically triggered anger could influence political behaviour. For example, anger triggered in one situation leads participants to attribute blame to others in a different situation even though it has nothing to do with the cause of the anger^13^. More generally, it has been shown that feelings affect our judgements in all areas of life. For example, a positive mood evoked by recalling happy memories influences judgments on unrelated topics such as whether a suspect in a hypothetical scenario is guilty or not^14^. Similarly, the influence of emotions on judgements has been demonstrated with positive and negative moods evoked by listening to music, experiencing sunny or rainy weather^15^, or by receiving performance feedback on unrelated task^16^. A vast body of literature shows that cognition is affected by both: integral emotions, i.e. resulting from or relevant to the judgement itself, as well as incidental emotions, not related to the given judgement or task^17^.

In the present study, we aimed to test whether the political consequences of anger can also be observed when the anger is not related to politics per se. More precisely, we investigate whether incidental anger, i.e., not politically specific anger, can shift individuals’ preferences for political leaders. To do so, we asked participants to select who they would vote for when shown pairs of faces, an experimental measure of political preferences that has been shown to vary with participants’ political orientation^12,18,19^. In particular, people who prefer stronger leaders in such leader choice tasks also show stronger agreements with the statement: “I think having a strong leader who does not have to bother with parliament and elections is a good thing”^19^.

Theorizing about ‘strong’ or dominant leaders has a long history in both political science and political psychology. Since Adorno’s^20^ influential work on authoritarian personality and more recent research on people’s leader preferences^21^, strong leaders are associated with perceptions of dominance. This is consistent with research in social psychology according to which dominance constitutes one of the two basic dimensions of person perception with warmth (or valence) as the other major dimension^22^. In relation to leader preferences, it has been shown that impressions of dominance are formed on the basis of cues related to their face, their voice, their behavior in non-leadership contexts and their political positions (for a review see^21^). For example, leaders with more masculine faces, with lower pitched voices, with more assertive and self-interested dispositions and with more right-wing policy positions are viewed as more dominant. Moreover, facial cues that inform social judgements, are sensitive to environmental factors and reflect actual political preferences^23–26^. In line with the idea that authoritarianism increases in contexts of social and/or economic threat^23^, recent studies have shown how external threats can bias political preferences towards more dominant looking leaders^27^ and authoritarianism in general^28^. Importantly, compared to more explicit measures, such as self-reported political opinions, political preferences from faces have been reliably shown to be sensitive to experimental manipulations. For instance, wartime and social threat scenarios consistently shift individuals’ leader preferences towards more dominant and masculine leaders in these tasks^23,27,29^.

To induce incidental anger in experimental settings we adapted an “Impossible Task”^30^, in which participants were asked to solve an anagram task where half of the anagrams are insoluble. Given the association between anger generated in a political context and authoritarianism, our hypothesis was that anger, even if not politically related, would bias people towards choosing stronger leaders.

We conducted two experiments investigating the effect of anger on the leader choices. Experiment 1 was exploratory, and Experiment 2 was preregistered and conducted on a larger sample.

## Experiment 1

### Methods

#### Participants

Participants were recruited through the online platform Prolific. Due to the nature of the anger induction involving a language task, we restricted our sample to native English speakers. 199 participants completed the experiment. We excluded participants who failed more than one attention check, resulting in a final sample of 194 participants (age: M = 36 ± 12 s.d.; 113 women; Table 1) which was used for the analysis. All participants gave informed consent and were paid £2 for their participation. The experiment and procedures were approved by the Royal Holloway, University of London Ethics Committee, and the experiment was performed in accordance with relevant guidelines and regulations.

**Table 1:** Descriptive statistics of the participant samples of Experiments 1 and 2.

	Experiment 1	Experiment 2	Statistical difference
	194	647	
Age	35.62 ± 11.78 [18–42.5]	35.64 ± 12.57 [25–44]	t(839) = − 0.02, p > .250
Gender	113 women, 80 men	371 women, 275 men	*X*^2^ (1, N = 839) = 0.04, p > .250
Anger	2.27 ± 1.23 [1–3]	2.30 ± 1.22 [1–3]	t(839) = − 0.27, p > .250
Anxiety	2.82 ± 1.36 [2–4]	2.82 ± 1.28 [2–4]	t(839) = 0.05, p > .250
Attentiveness	4.33 ± 0.82 [4, 5]	4.33 ± 0.84 [4, 5]	t(837) = 0.18, p > .250
Political orientation	4.83 ± 1.46 [4–6]	4.66 ± 1.53 [4–6]	t(823) = 1.37, p = .172
Authoritarianism	− 1.38 ± 1.59 [− 2.63 to − 0.33]		
Alexithymia	51.12 ± 8.54 [45–57]	53.17 ± 9.34 [46–59]	t(839) = − 2.72, p = .007
Baseline leader preference	0.35 ± 0.11 [0.27–0.41]	0.36 ± 0.12 [0.27–0.42]	t(839) = − 0.79, p > .250
Post anger induction leader preference	0.35 ± 0.12 [0.25–0.42]	0.36 ± 0.15 [0.24–0.44]	t(839) = − 0.99, p > .250

Mean values are presented with the standard deviation and inter-quartile range [first quartile–third quartile]. T-values were obtained using Student’s t-tests.

#### Stimuli

**Figure 1:**
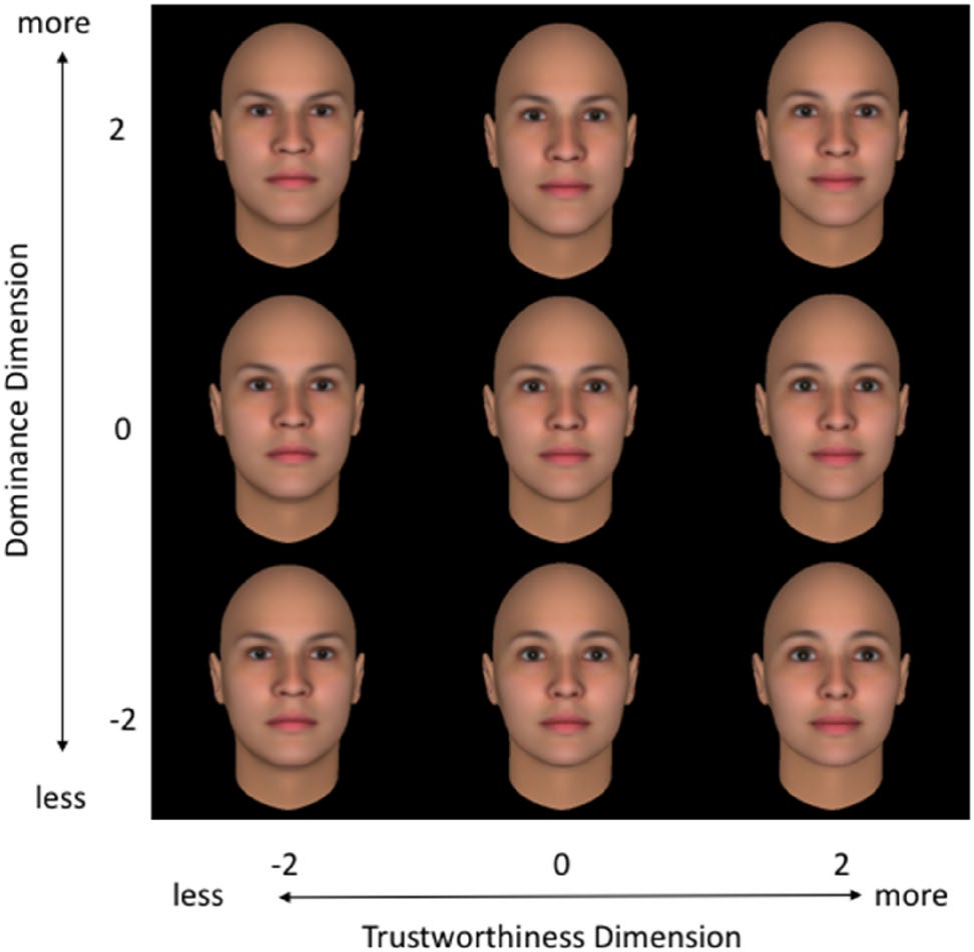
Stimuli in the leader choice task. The faces varied along two dimensions: Trustworthiness (X-axis) and Dominance (Y-axis), in a range of − 2 to + 2 points with an increment of 2 points.

As stimuli in the leader choice task, we used eight non-gendered computer generated faces, created in FaceGen 3.1 (Singular Inversions) software and controlled for their level of trustworthiness and dominance^22^. These stimuli have been successfully used in previous studies to elicit dominance and trustworthiness judgments both at the explicit and implicit level^19^. Each face was characterized by two dimensions: dominance and trustworthiness in a range of − 2 to + 2 points with an increment of two points. The stimuli included every combination of dominance and trustworthiness in this range resulting in nine faces (Fig. 1). Stimuli were always presented in pairs in which the faces were 2 to 4 points different from each other on at least one dimension (dominance or trustworthiness). This resulted in 36 pairs of faces.

#### Procedures

The experiment was designed in Gorilla Experiment Builder (www.gorilla.sc; Anwyl-Irvine, Massonnié, Flitton, Kirkham & Evershed, 2019). The experiment consisted of a leader choice task presented twice: before and after the anger induction. As previous studies using a similar leader task did not report any retest effects, a pre-test post-test design was adopted^19,32^.

In the leader choice task, each trial began with a central fixation cross presented for 250 ms. Then, two faces were presented simultaneously: one on each side of the fixation cross. Participants had to indicate who they would vote for in a hypothetical national election by clicking on the selected face. Faces were presented for 3 s and participants were instructed to follow their first impression. During the initial instructions, participants were informed that if they do not answer within 3 s, the next trial will be automatically presented. Participants also received two practice trials. During the experiment, the 36 possible pairs of faces were presented in a random order. The position of the faces was counterbalanced with the more dominant and the more trustworthy faces appearing equally often on the left or on the right. To make sure that participants were engaged with the task, two attention check trials were introduced in which two identical faces were presented, one of those with an instruction “select this face”. Including these attention checks the leader choice task consisted of 38 trials and took approximately 2 min to complete. The analysis showed that in our experiment, participants missed on average 1.2 trials in the leader choice task (s.d. = 2.3).

To induce anger, we adapted the paradigm of “Impossible Task”^30,32^ in which participants are asked to perform a task in settings that render a positive outcome impossible to solve problems with no solution. Here, we designed an anagram task where participants had to form words out of scrambled strings of letters with more than half anagrams insoluble. Similar tasks have previously shown to successfully induce anger and frustration in previous studies, both in the lab and online. Anagrams were taken from previous studies^33,34^. All consisted of 5 letters. In total, there were 13 soluble anagrams (e.g. dhilc = child, ssoia = oasis), and 15 impossible ones (e.g. igdrt). On each trial, participants saw an anagram presented on a screen and a response box below where they could type the solution. The screen was presented for 10 s, if they did not answer within the time limit, a feedback stating “Too slow” was presented for 500 ms and the next trial started. At the end of the anagram task, regardless of their performance participants always saw the same feedback saying “You scored below average” paired with a sad emoji face. This task took approximately 6 min to complete. During the anagram task, two attention checks were introduced, namely two anagrams that already formed an existing word. At the beginning of the task, participants were instructed to simply copy the word they see on those trials. In total, six attention checks were introduced during all parts of the task to make sure that participants were engaged with the task throughout the experiment.

At the very end of the experiment, participants were asked to rate their current emotional state (anger, anxiety, attentiveness) on a scale from 1 (not at all or very slightly) to 5 (extremely) answering three questions: *How angry/ anxious/ attentive were you in the last minutes?* Toronto Alexithymia Scale (TAS^35^) was also administered given the role that this subclinical trait plays in emotional experience, as well as Very Short Authoritarianism^36^ (VSA) scale. Finally, participants were asked to indicate their political affiliation on a scale from 1 (very conservative) to 7 (very liberal; Table 1).

#### Analysis

As we were interested in the effect of anger on the shift towards stronger leaders, our main dependent variable was the change in the probability of choosing more dominant and less trustworthy looking leaders after the anger induction relative to the pre-manipulation phase (ProbPost–ProbPre) obtained through the following steps: First, based on their choices on the 38 trials, we modelled leader choices for each participant separately by running a generalized linear mixed-effects model with face position (left/right) and the order of presentation as random factors, and the faces’ levels of trustworthiness and dominance as fixed effects. On the basis of coefficients obtained from this model the predicted probability of choosing a more dominant and less trustworthy (i.e., a 1-point more dominant and 1-point less trustworthy) leader was calculated. This thus gives us two data points for each participants: one corresponding to the predicted probability of choosing a more dominant and less trustworthy leader before the anger induction and one after the anger induction. Our key dependent variable, the change in the predicted probability of choosing more dominant and less trustworthy leader, was calculated as a subtraction of these leader choice probabilities obtained before and after anger induction. Analysis were conducted in R software.

### Results

#### Baseline leader preferences

We first analyzed participants’ leader preferences before anger induction (the completion of the anagram task). Replicating previous studies on leader preferences^19,23^, participants were significantly less likely to prefer the strong leader in this baseline condition (M = 0.35 ± 0.11 s.d.; Student’s t-test against chance: t(193) = − 18.93, p < 0.001). In addition, in line with the literature on leader choice^18,37^, a significant effect of participants’ political affiliation was found such that participants reporting to be more conservative were more likely to prefer the strong leader (ß =− 0.02 ± 0.01 s.e.m., t(192) = − 2.23, p = 0.027; this association was stable after controlling for age and gender: ß = − 0.02 ± 0.01 s.e.m., t(189) = − 2.153, p = .033).

#### Change in leader preferences

**Figure 2:**
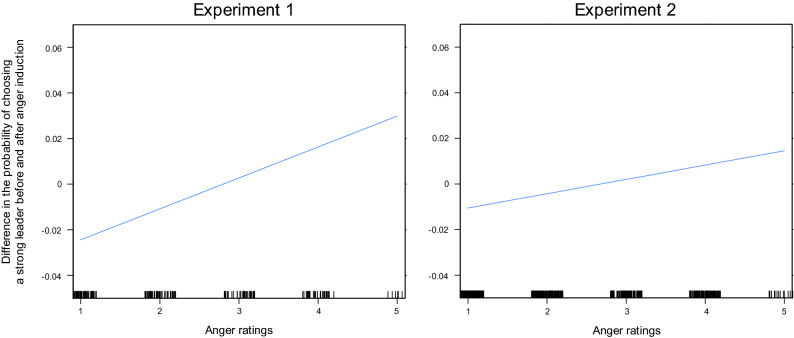
Association between experienced anger and change in leader preferences in Experiments 1 and 2. *Left panel:* The relationship between the dependant variable (ProbPost–ProbPre) and anger ratings estimated by the full regression model (with anger, anxiety, attentiveness, political affiliation and TAS as predictors) for the two experiment is plotted together with the 95% confidence intervals (Left: Experiment 1; Right: Experiment 2).

We then examined the change in probability of choosing a stronger leader after anger induction as a function of experienced anger. In line with our hypothesis, one-way linear regression taking self-reported anger as the only predictor revealed that higher levels of anger were associated with an increase in the preference for strong leaders following anger induction (ß = 0.01 ± 0.01 s.e.m., t(192) = 2.10, p = 0.037, model adjusted R^2^ = 0.017; Table 2). This effect was robust to the inclusion of the other self-reported emotional states (anxiety and attentiveness), political attitudes (political orientation and authoritarianism), alexithymia score and demographic variables (age and gender) as covariates (ß = 0.02 ± 0.01 s.e.m., t(182) = 2.14, p = 0.033; Table 2, Fig. 2). By contrast, no significant effect of self-reported experienced anxiety was evidenced (ß = − 0.00 ± 0.01 s.e.m., t(183) = − 0.48, p > 0.250), suggesting a specific association between the change leader preferences and experienced anger.

**Table 2:** Results of the regression analyses on the change in the preference for a strong leader after the anger induction in Experiments 1 and 2.

	One-way regression			Full model		
	Experiment 1	Experiment 2	Meta-analysis	Experiment 1	Experiment 2	Meta-analysis
Intercept	− 0.01 ± 0.01 t(192) = − 1.00	− 0.00 ± 0.00 t(645) = − 0.72	− 0.00 ± 0.00 z = − 1.15	0.00 ± 0.02 t (182) = 0.01	− 0.01 ± 0.01 t(622) = − 1.05	− 0.01 ± 0.01 z = − 0.90
Anger	0.01 ± 0.01 t(192) = 2.10 *	0.01 ± 0.00 t(645) = 2.84 *	0.01 ± 0.00 z = 3.52 ***	0.02 ± 0.01 t(182) = 2.14 *	0.01 ± 0.01 t(622) = 1.56	0.01 ± 0.00 z = 2.36 *
Anxiety				− 0.01 ± 0.01 t(182) = − 0.66	0.00 ± 0.01 t(622) = 0.95	0.00 ± 0.01 z = 0.37
Attentiveness				− 0.01 ± 0.01 t(182) = − 0.99	− 0.01 ± 0.00 t(622) = − 2.48*	− 0.01 ± 0.00 z = − 2.63**
Political orientation				0.02 ± 0.01 t(182) = 1.73°	0.00 ± 0.00 t(622) = 0.03	0.01 ± 0.01 z = 0.79
Authoritarianism				0.01 ± 0.01 t(182) = 1.37		
Alexithymia score				− 0.01 ± 0.01 t(182) = 0.62	− 0.00 ± 0.00 t(622) = − 0.14	− 0.00 ± 0.00 z = − 0.55
Age				0.00 ± 0.01 t(182) = 0.62	− 0.01 ± 0.00 t(622) = − 1.79	− 0.00 ± 0.01 z = − 0.50
Gender				− 0.01 ± 0.01 t(182) = − 0.35	0.01 ± 0.01 t(622) = 0.91	0.01 ± 0.01 z = 0.60
N	194	647		191	630	

The first line corresponds to the standardized regression coefficient, presented with the standard errors to the mean. N corresponds to the number of participants included in each regression (without missing data). ***indicates a p-value < .001, **indicates a p-value < .010, *indicates a p-value < .050 and °indicates a p-value < .100.

Finally, although self-reported experienced anger at the end of the experiment was not significantly associated with differences in leader preferences before anger induction (ß = 0.00 ± 0.01 s.e.m., t(189) = 0.04, p > 0.250), we conducted additional analyses to account for potential carry-over effects. Regression analyses on leader preferences after the anger induction task taking baseline leader preference as control variable confirmed that participants who reported higher levels of anger were more likely to prefer stronger leaders after anger induction (without controlling for additional individual variables: ß = 0.02 ± 0.01 s.e.m., t(188) = 2.24, p = 0.027; and after having included all the control variables: ß = 0.02 ± 0.01 s.e.m., t(181) = 2.18, p = 0.031).

Although previous studies using the same leader choice task did not reveal any retest effect^19,31^, the pre-post nature of our experiment design still creates the possibility that the results we obtained are not related to the induction of anger but only to the repetition of the task. Nonetheless, three elements provide evidence that the reported effect is truly related to incidental anger and not simply to a test–retest effect. First, apart from the differences associated with experienced anger, leader preferences after anger induction were not significantly different from leader preferences before the anger induction (ß = − 0.01 ± 0.07 s.e.m., t(189) = − 1.05, p > 0.250), suggesting that participants’ level of anger is genuinely driving the reported effect. Second, reaction time analyses suggest that the faces presented in the leader choice task were processed similarly before and after anger induction (both p-s > 0.216; see Supplementary Table 1). Finally, one could argue that anger, rather than shaping leader preferences, changes the visual perception of presented faces. If this explanation was correct, it could be possible that after the anger induction, individuals’ true leader preferences would stay the same, but their measured preferences would be seen to shift because participants perceive the face stimuli differently, i.e. faces appear less dominant and more trustworthy. In line with this hypothesis, previous research showed that the emotional state of the viewer affects perception of emotions in faces^38^. We therefore conducted a supplementary experiment on a new sample of participants (N = 208) who were asked to indicate which of the two faces belonged to the more successful individual, as a means of assessing the effect of anger induction on a different domain of social perception. This experiment revealed no significant difference in participants’ preferences associated with self-reported anger after the same experimental manipulation (ß = 0.00 ± 0.01, t(198) = 0.52, p > 0.250; see Supplementary information for details).

Consistent with our predictions, the results of Experiment 1 thus suggest that incidental anger is associated with an increase in the probability of choosing a strong leader.

## Experiment 2

Experiment 2 aimed to replicate the findings of Experiment 1 on a larger sample of participants. We preregistered our design, analysis and hypothesis for the effect of self-reported experienced anger on the difference in leader preferences before and after the anagram task (link removed to ensure double-blind reviewing).

### Methods

#### Participants

The power analysis in G*Power 3.1 showed that to detect an effect size *R*^2^ = 0.02 in our model 647 participants are required. Participants were recruited through Prolific. At one point during data collection a server failure occurred; we excluded all the data collected during that time. 661 participants completed the experiment. Consistent with our pre-registration, we excluded those participants who failed more than one attention check, resulting in the targeted final sample of 647 individuals included in the analysis (age: M = 35.6 ± 12.6 s.d.; gender: 371 women; Table 1). All participants gave informed consent and were paid £1.75 for their participation. The experiment and procedures were approved by the Royal Holloway, University of London Ethics Committee, and the experiment was performed in accordance with relevant guidelines and regulations.

#### Stimuli and procedures

Stimuli and procedures were identical as in Experiment 1.

#### Analysis

All analyses were the same as in Experiment 1.

### Results

#### Baseline leader preferences

As in Experiment 1, we first analyzed leader preferences before anger induction. We replicated participants’ baseline preference for less strong leaders (M = 0.36 ± 0.12 s.d.; Student’s t-test against chance: t(646) = − 29.03, p < 0.001) as well as the expected significant association between participants’ self-reported political affiliation and the preference for strong leaders (ß = − 0.02 ± 0.01 s.e.m., t(629) = − 3.361, p < 0.001; after controlling for age and gender: ß = − 0.02 ± 0.01 s.e.m., t(626) = − 3.14, p = 0.002). Finally, as in Experiment 1, no significant association between self-reported anger at the end of the experiment and leader preferences before anger induction was found (ß = 0.01 ± 0.01 s.e.m., t(645) = 1.251, p = 0.211).

#### Change in leader preferences

The association between self-reported anger and the change in leader preferences after anger induction was also significant in this second experiment (ß = 0.01 ± 0.00 s.e.m., t(628) = 2.57, p = 0.010; model adjusted R^2^ = 0.011; Table 2). However, this effect was no longer statistically significant after the inclusion of self-reported anxiety and attentiveness, political affiliation, alexithymia score, gender and age as predictors (ß = 0.01 ± 0.01 s.e.m., t(622) = 1.56, p = 0.121; Table 2, Fig. 2). Once again, no significant effect of self-reported experienced anxiety was evidenced (ß = 0.01 ± 0.01, t(622) = 0.95, p > 0.250).

As for Experiment 1, we conducted regression analyses on leader preferences after anger induction taking baseline leader preferences as predictor to control for potential carry-over effects. These analyses confirmed the significant association between self-reported anger and leader preferences after anger induction (ß = 0.01 ± 0.00 s.e.m., t(644) = 3.11, p = 0.002) although this effect was only found as a trend after the inclusion of control variables (ß = 0.01 ± 0.01, t(621) = 1.82, p = 0.069).

Finally, analyses on reaction times confirmed that the way participants processed the stimuli was similar before and after anger induction (both p-s > 0.203; see Supplementary Table 1) suggesting that the evidenced association between incidental anger and the change in leader preferences was not due to a mere test–retest effects.

Following the Open Science Collaboration’s recommendations (2012), we conducted a fixed-effects meta-analysis to determine the overall evidence that Experiment 1 and 2 provide for the change in leader preferences following anger induction (using R metaphor package^39^). Across both experiments, we calculated the overall effect sizes (beta coefficients) of self-reported anger in the all the regression models we conducted. All these analyses revealed a significant association of between self-reported anger and the difference in leader preferences before and after anger induction, providing evidence of the robustness of the evidenced effect (see Table 2). Consistent with the results of Experiment 1, these findings suggest that higher levels of incidental anger were associated with a preference for strong leaders.

## General discussion

The goal of our studies was to investigate the association between incidental anger and a preference for strong leaders. Based on the important literature showing a preference for stronger leaders following threat-related experimental manipulations as well as its link with self-reported political conservatism and authoritarianism^19,23,27,29,40,41^, we operationalized the preference for strong leaders as the preference for dominant and untrustworthy faces in the leader choice task. Consistent with our predictions, we found that the degree to which people experienced anger increased the probability of choosing more dominant and less trustworthy faces, suggesting that non-politically generated incidental anger is sufficient for increasing the preference for strong leaders.

The findings of Experiments 1 and 2 may be specific to the context of political leadership, as our Supplementary study showed that anger does not affect judgements of non-political individual successfulness. (see Supplementary Material). We also found that anxiety ratings did not predict an increase preference for a stronger leader. This is consistent with the previous findings showing that anger generated in a political context affects political decision making differently than fear or anxiety^7–11^. Importantly, across the two experiments, it was the degree of experienced anger and not experienced anxiety that predicted the change in leader choice. In line with recent influential views of constructed emotions (Barrett, 2017), emotion induction involves two parts: affect induction plus meaning making. Our findings suggest that the change in leader preferences is affected by the meaning (i.e. anger as opposed to anxiety) that participants assigned to the induced affect that our manipulation intended to elicit.

At this stage, one could wonder about the precise psychological mechanisms that may link incidental anger and preference for strong leaders. A first possibility is that anger makes people to temporarily operate as in an aggressive state, similar to those found in situations of war and conflict. Previous studies showed that threatening or war scenarios are associated with a preference for taller, more masculine, more dominant and less trustworthy leaders^12,19,29^. Furthermore, Laustsen and Petersen^12^ found increased preference for dominant leaders only in participants for whom the primary response to conflict was anger. Based on this, it has been suggested^21^ that preferences for dominant leaders are higher among those with “conflict mindsets”. The conflict mindset may be a personality trait but also a temporary state induced by affective experiences which does not need to be initially politically motivated, as suggested by our results.

Another possibility is linked to the stimuli used in this experiment. Our stimuli were based on the models of social perception developed by Oosterhof and Todorov^22^ who found that the more extreme faces on the trustworthiness dimension were also systematically perceived as angrier. This suggests a possible explanation for our findings: angry people want angry leaders who can represent them. As Donald Trump told CNN: “I’m angry, and a lot of other people are angry, too, at how incompetently our country is being run. (..) Anger and energy is what this country needs” (CNN transcripts^42^). This explanation is consistent with theories stating that people choose leaders based on the “being one of us” criterion^43^. Further, our results suggest that this process may operate in a state-like way and independently of the political context, i.e. based on transient changes in experienced emotions which are not linked to political events^44^.

Our results highlight the role of anger in political preferences. Although early theories viewed preferences for strong leaders as a stable characteristic of authoritarian personalities^20^, research in political psychology highlighted that leader preferences are flexible and context dependent^21^. Adding to the findings of Laustsen and Petersen^12^ who showed that integral anger plays an important role in shaping leader preferences, here we showed that incidental anger, not related to the leader choice task is also associated with a preference for strong leaders. By highlighting the influence of non-politically motivated emotions on political behaviour, our findings may contribute to ongoing cross-disciplinary debates about the role that affect and emotional experiences may influence political behaviour^45^.

However, three main limitations of our work should be noted. First, although we contrasted it with anxiety, it is not possible to know to what extent the observed effect on leaders' preferences is specific to incidental anger. Indeed, other emotions, such as feelings of helplessness, may have been triggered by our experimental manipulation and influenced our results. Second, because we did not measure anger experienced prior to anger induction, it is difficult to disentangle the role of integral anger and incidental anger in explaining our results. An alternative explanation for our results is that our experimental manipulation triggered differences in leader preferences only in individuals who had high levels of integral anger, as suggested by models highlighting the importance of predisposition to understand political responses to crises^46–48^. Further research should therefore be conducted to test the specific role of trait and state anger on the political response to acute events.

The literature on political emotions and their influence on people’s political decisions and behaviour is vast^49^ and it has been argued that different emotions may have distinct political consequences. In the present study, we set out to investigate in a rigours and replicable way the specific effect that non-political anger has on political leader choices, rather than to contrast the effect of anger relative to other emotions. Therefore, we do not wish to make any claims as to whether anger only or that anger mainly is linked to such political preferences as described in our studies. Future research could investigate the distinct and contrasting effects that different emotions may have on political leader choices.

Finally, because of its sensitivity to experimental manipulation, we used a well-validated task based on first impressions of faces to study the effect of apolitical anger on leaders' preferences. However, while this experimental protocol provides insight into the type of traits that may be valued in leaders in different contexts, other factors such as political affiliation play a more central role in real elections, sometimes interacting with the perception of candidates' personality traits^21,50^. Nevertheless, our results suggest that emotional factors, in addition to well-identified contextual factors such as war and social threats, can influence our preferences for particular leaders. Further research should therefore examine what emotions, besides anger, may influence leader choice when not generated by a political context.

To conclude, the results presented in these experiments show that anger that is not generated in a political context can shift political preferences in specific directions. This finding is relevant for a large body of recent research across political psychology and political science on the role of emotions in politics^51^, and in particular for new directions that attempt to bridge physiological states^52^ with experienced affect^53^ during political decision making.

## Supplementary Information

Supplementary Information.
