# Supplementary material for: Non-political anger shifts political preferences towards stronger leaders
Source: Sci Rep. 2022 Jul 11;12:11766. doi: 10.1038/s41598-022-15765-8 (PMC9273584; doi:10.1038/s41598-022-15765-8)
Supplement: Supplementary file 1 — Supplementary Information. [file 41598_2022_15765_MOESM1_ESM.docx]

**Non-political anger shifts political preferences towards stronger leaders**

Klaudia B. Ambroziak^1^, Lou Safra^2^ and Manos Tsakiris^1, 3,^

**Supplementary analyses**

Attentiveness and reaction time analyses

To assess the potential effects of attentiveness on participants’ responses to the voting tasks, we conducted, for each experiment and for each voting task, a mixed linear regression on participants’ reaction times with participants’ ID as a random factor and attentiveness scores as well as the absolute differences in trustworthiness and in dominance between each pair of avatar as fixed effects. The statistics were computed using R lmerTest package which uses Satterthwaite's denominator degrees of freedom to assess the statistical significance of mixed linear regressions’ fixed effects.

This analysis confirmed that participants were sensitive to the avatars’ dominance and trustworthiness with choices being faster for more dissimilar pairs (all *p*-s < .003 except for the difference in dominance for the post-anger task of Experiment 1, *p* = .08). No significant effect of reported attentiveness on the sensitivity to the avatars’ dominance or trustworthiness was evidenced for any of the experiment, suggesting that participants reporting being less attentive did not completed the voting tasks more randomly (all *p*-s > .250, except for the interaction between the difference in trustworthiness and attentiveness scores in the pre-anger task of Experiment 1, *p* = .058). These results were further confirmed by a meta-analysis conducted on the three experiments (Table S1).

|  | Estimate | Standard error | z-value | p-value |
| --- | --- | --- | --- | --- |
| Meta-analysis – voting task before the anger induction | | | | |
| Intercept | 1651.57 | 26.12 | 63.22 | < .001 |
| \|∆Dominance\| | -12.56 | 1.50 | -8.37 | < .001 |
| \|∆Trustworthiness\| | -23.30 | 1.50 | -15.50 | < .001 |
| Attentiveness | 31.67 | 14.17 | 2.24 | .025 |
| Attentiveness*\|∆Dominance\| | -1.52 | 1.50 | -1.01 | > .250 |
| Attentiveness*\|∆Trustworthiness\| | -2.00 | 1.63 | -1.22 | > .250 |
| Meta-analysis – voting task after the anger induction | | | | |
| Intercept | 1507.59 | 11.07 | 136.25 | < .001 |
| \|∆Dominance\| | -11.59 | 2.66 | -4.35 | < .001 |
| \|∆Trustworthiness\| | -18.50 | 2.18 | -8.47 | < .001 |
| Attentiveness | 42.57 | 11.06 | 3.85 | < .001 |
| Attentiveness*\|∆Dominance\| | -1.54 | 1.46 | -1.06 | > .250 |
| Attentiveness*\|∆Trustworthiness\| | -0.68 | 1.46 | -0.47 | > .250 |

Table S1 – Meta-analyses of the reaction time results for the voting tasks completed before and after the anger induction for all the experiments

1. **Experiment 3**

In Experiment 3, we investigated whether experienced anger affects other types of judgements related to leadership abilities. Using a task identical as in Experiments 1 and 2, we asked participants to judge which of the faces belonged to the more successful individual. Previous research suggests that people choose leaders based on perceived level of individual success (Grabo, Spisak & van Vugt, 2017) and perceived competence, even without any political information, is the best predictor of election outcomes (Todorov, Mandisodza, Goren, & Hall, 2008). Based on this research, we predicted that anger would shift judgements of most successful individuals towards more dominant and less trustworthy faces, as in the leader choice task. We preregistered our design, analysis and hypothesis (link removed to ensure double-blind reviewing).

**4.1 Methods**

**Participants**

208 participants took part in Experiment 3. Consistent with preregistration, we excluded participants who failed more than one attention check. Data from the final sample of 200 participants (age: M = 34.7, SD = 11.9; gender: 126 females, 1 unknown) was included in the analysis. All participants gave informed consent and were paid £1.5 for their participation. The experiment and procedures were approved by the Royal Holloway, University of London Ethics Committee, and the experiment was performed in accordance with relevant guidelines and regulations.

**Stimuli and procedures**

Stimuli and procedures were the same as in Exp 1-2 but this time instead of asking the participants to indicate a person they would vote for, we asked which face appeared as the most successful individual. During initial instructions, it was explained to the participants that by successful we mean someone "who is able to achieve her or his goals by herself/himself." This concept was aimed to refer to general competence and not politically-centred one.

**Analysis**

Analyses were the same as in Experiment 1 and 2.

**4.2 Results**

As in Exp 1 and 2, we conducted a multiple linear regression analysis across participants with ProbPost – ProbPre as dependent variable and with anger ratings (on 1-5 scale) as the main predictor and with anxiety, attentiveness, political affiliation and TAS as additional predictors. This regression model was not significant: adjusted *R2* = -0.01, *F*(5,181) = 0.71, *p* = 0.6. Unlike in Experiment 1 and 2, ANOVA showed no effect of anger *F*(1,181) = 0.00, *p* = 0.997. The difference in probability of choosing more dominant and less trustworthy face as more successful did not change as a function of anger ratings (see Figure 5). Other predictors were also not significant (see Table 3).

Since previous research suggested that people based their leader choices on perceived level of individual success (Grabo et al, 2017), we expected that anger will shift choices in the present task towards more dominant and less trustworthy faces similarly as in Experiment 1 and 2. Contrary to our predictions, these results show that anger induction did not affect the perception of the individual characteristics that are necessary to ensure individual success suggesting that the results of Experiments 1 and 2 seem to be specific to the context of political leadership. This finding also suggests that anger does not change the visual perception of presented faces but rather it seems specific to the judgment in the leader choice task.


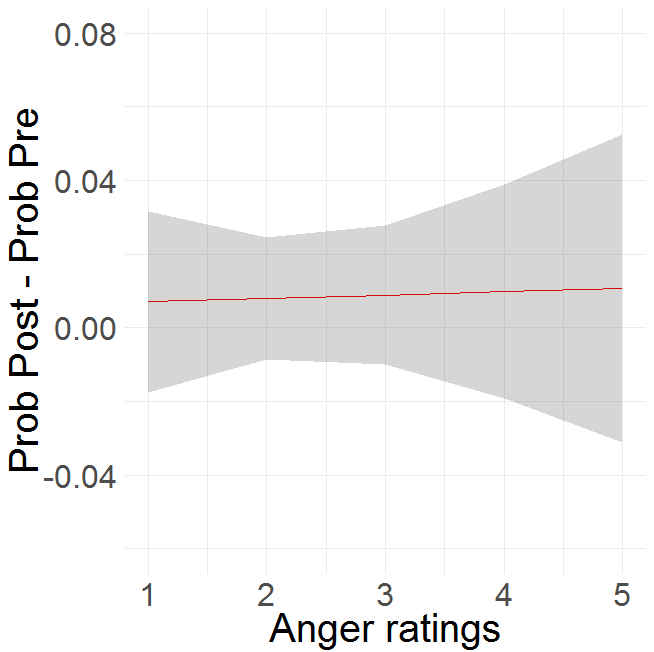

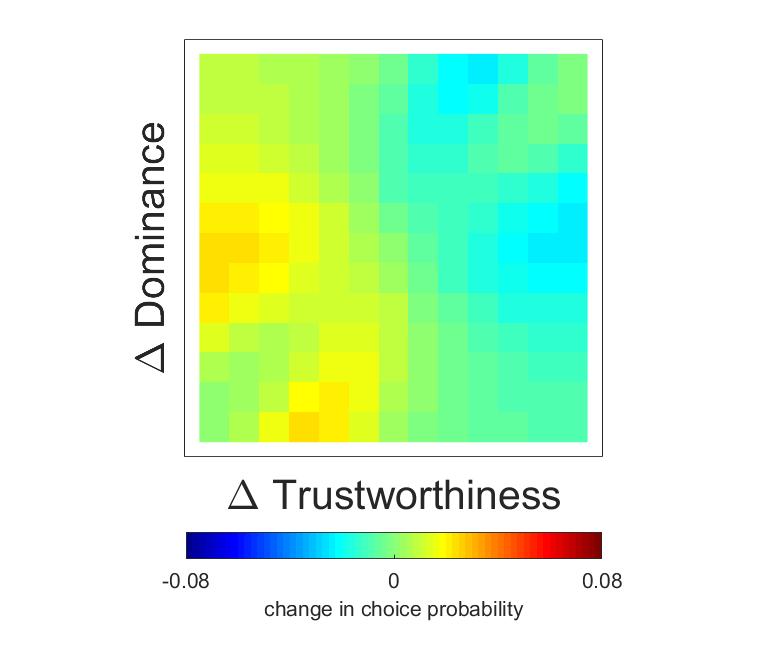


**Figure S1.** Results of Experiment 3. *Left panel:* there was no relationship between the dependant variable (ProbPost – ProbPre) and anger ratings. *Right panel:* A heatmap for the probability of choosing a face as more successful according to Trustworthiness (X- Axis) and Dominance (Y-Axis). Values are changes is probabilities as a difference of Post-Pre anger induction between higher and lower anger participants. Note that this median split between higher and lower anger was used here only for display purposes, give that in our statistical analysis anger was entered as a continuous variable.

| ANOVA Table | | | | | |
| --- | --- | --- | --- | --- | --- |
| Response: ProbPost - ProbPre | | | | | |
|  | Df | SS | MS | F value | p |
| Anger | **1** | **0.00** | **0.00** | **0.00** | **0.997** |
| Anxiety | 1 | 0.01 | 0.01 | 1.06 | 0.303 |
| Attentiveness | 1 | 0.02 | 0.02 | 1.28 | 0.260 |
| Political Orientation | 1 | 0.01 | 0.02 | 0.62 | 0.434 |
| TAS | 1 | 0.01 | 0.02 | 0.57 | 0.452 |
| Residuals | 181 | 2.22 | 0.01 |  |  |

**Table S2:** Results of Experiment 3: ANOVA
